# Differentiating between the possibility and probability of SARS-CoV-2 transmission associated with wastewater: empirical evidence is needed to substantiate risk

**DOI:** 10.1093/femsmc/xtab007

**Published:** 2021-05-04

**Authors:** Warish Ahmed, Kyle Bibby, Patrick M D'Aoust, Robert Delatolla, Charles P Gerba, Charles N Haas, Kerry A Hamilton, Joanne Hewitt, Timothy R Julian, Devrim Kaya, Paul Monis, Laurent Moulin, Colleen Naughton, Rachel T Noble, Abhilasha Shrestha, Ananda Tiwari, Stuart L Simpson, Sebastien Wurtzer, Aaron Bivins

**Affiliations:** CSIRO Land and Water, Ecosciences Precinct, 41 Boggo Road, QLD 4102, Australia; Department of Civil & Environmental Engineering & Earth Sciences, University of Notre Dame, 156 Fitzpatrick Hall, Notre Dame, IN 46556, USA; Department of Civil Engineering, University of Ottawa, Ottawa, ON, Canada; Department of Civil Engineering, University of Ottawa, Ottawa, ON, Canada; Department of Environmental Science, Water and Energy Sustainable Technology Center, University of Arizona, 2959 W. Calle Agua Nueva, Tucson, AZ 85745, USA; Drexel University, Philadelphia, PA, USA; School of Sustainable Engineering and the Built Environment and the Biodesign Institute Center for Environmental Health Engineering, Arizona State University, Tempe, AZ 85287, USA; Institute of Environmental Science and Research Ltd (ESR), Porirua, 5240, New Zealand; Eawag, Swiss Federal Institute of Aquatic Science and Technology, Dübendorf CH-8600, Switzerland; School of Chemical, Biological, and Environmental Engineering, Oregon State University, 105 SW 26th St #116, Corvallis, OR 97331, USA; South Australian Water Corporation, Adelaide, Australia; Eau de Paris R&D Laboratory. 33 Av. Jean Jaures 94200 Ivry/seine, France; University of California Merced Department of Civil and Environmental Engineering, 5200 N, Lake Rd. Merced, CA 95343, USA; University of North Carolina Institute of Marine Sciences, Morehead City, NC, USA; Division of Environmental and Occupational Health Sciences, School of Public Health, University of Illinois Chicago, Chicago, IL, USA; Finnish Institute for Health and Welfare, Expert Microbiology Unit, Kuopio, Finland; CSIRO Land and Water, Lucas Heights, NSW 2234, Australia; Eau de Paris R&D Laboratory. 33 Av. Jean Jaures 94200 Ivry/seine, France; Department of Civil & Environmental Engineering & Earth Sciences, University of Notre Dame, 156 Fitzpatrick Hall, Notre Dame, IN 46556, USA

People infected with severe acute respiratory syndrome coronavirus 2 (SARS-CoV-2) shed the virus and its genetic material via their sputum, nasopharyngeal secretions, saliva, urine and feces (Cevik *et al*.^2021^). Hence, public health and water quality scientists throughout the world have been monitoring untreated and/or primary treated wastewater and sludge for the surveillance of SARS-CoV-2 in communities (https://arcg.is/1aummW). Numerous reviews have discussed the possibility of SARS-CoV-2 transmission to humans from exposure to wastewater or waters receiving untreated or inadequately treated wastewater based on limited empirical evidence (Adelodun *et al*. 2020; Bilal *et al*. 2020; Olusola-Makinde and Reuben 2020; Elsamadony *et al*. 2021; Khorram-Manesh, Goniewicz and Burkle 2021; Shutler *et al*. 2021). Multiple transmission routes have been suggested, including waterborne transmission, airborne transmission, contact with contaminated surfaces (fomites) and subsequent touching of mucous membranes such as the mouth, nose, or eyes. Herein, we briefly summarize the empirical evidence pertaining to the transmission of SARS-CoV-2 associated with wastewater exposure.

SARS-CoV-2 RNA has been detected at high concentrations in feces of COVID-19 patients (10^8^ genome copies (GC)/g) and COVID-19 patients are reported to shed RNA in their feces >30 days (Li, Wang and Lv 2020). In contrast, infectious SARS-CoV-2 is typically only isolated from nasopharyngeal specimens during the first nine days of infection (Cevik *et al*. 2021). Studies have reported the isolation of infectious SARS-CoV-2 from the feces and urine of COVID-19 patients (Sun *et al*. 2020; Xiao *et al*. 2020); however, this is rare. For example, Wang *et al*. (2020) screened 153 fecal samples and isolated infectious SARS-CoV-2 from only four specimens with ‘high copy numbers’. Wölfel *et al*. (2020) failed to detect infectious SARS-CoV-2 in 13 samples collected from four patients over six days. Another study found that infectious SARS-CoV-2 was rapidly (i.e. 5-fold within 1 h and loss of 80% viral infectivity after 24 h) inactivated by simulated colonic fluid (Zang *et al*. 2020). Similarly, during longitudinal studies of monkeys inoculated with SARS-CoV-2, infectious SARS-CoV-2 was isolated from feces two to seven days post-infection from one of six monkeys at concentrations four orders of magnitude lower than RNA (Woolsey *et al*. 2020). Collectively, the available data indicate that for each shedding route, infectious SARS-CoV-2 is shed for shorter durations and at lower prevalence and concentration than SARS-CoV-2 RNA.

SARS-CoV-2 RNA has been commonly detected at concentrations ranging from 20 to more than 10^6^ GC/L in untreated wastewater and >10^8^ GC/L in primary sludge (Ahmed *et al*. 2020a; Mlejnkova *et al*. 2020; Peccia *et al*. 2020). SARS-CoV-2 RNA has been detected in 25% of final treated effluent samples at concentrations ranging from 1.3 to approximately 10^5^ GC/L (Ampuero *et al*. 2020; Balboa *et al*. 2021; Carrillo-Reyes *et al*. 2021; Rimoldi *et al*. 2020; Saguti *et al*. 2021; Kumar *et al*. 2021a; Sherchan *et al*. 2020; Westhaus *et al*. 2021).

SARS-CoV-2 RNA has also been detected in environmental waters. For surface waters receiving untreated wastewater in areas with poor sanitation, RNA has been detected in 100% of samples (*n* = 18) at concentrations over 10^6^ GC/L (Guerrero-Latorre *et al*. 2020; Iglesias *et al*. 2020). Whereas for surface waters receiving treated wastewater, SARS-CoV-2 RNA has been collectively detected in 3/7 (43%) samples, but RT-qPCR quantification cycle (Cq) values or concentrations were not reported (Haramoto *et al*. 2020; Rimoldi *et al*. 2020). However, attempts to detect infectious SARS-CoV-2 from six untreated wastewater samples, four treated wastewater and six river water samples in Italy were not successful (Rimoldi *et al*. 2020). Westhaus *et al*. (2021) could not detect infectious SARS-CoV-2 in untreated and treated wastewater in Germany. Additionally, Desdouits *et al*. (2021) did not detect SARS-CoV-2 RNA in shellfish in 187 samples across 37 sites along the French coast between April and August 2020.

Inactivation and decay studies demonstrated that SARS-CoV-2 RNA persisted longer (*T*_90_ = 18–25 days; Ahmed *et al*. 2020b) than infectious viruses *(T*_90_ = 1.2–1.9 days; Bivins *et al*. 2020; de Oliveira *et al*. 2021; Sala-Comorera *et al*. 2021) when seeded in wastewater and surface water and seawater. This persistence differential leads to a decreasing ratio of infectious virus/RNA GC over time. For instance, during a 7-day period in seeded wastewater, the median tissue culture infectious dose (TCID_50_)/GC ratio decreased from 1 to 100 to less than 1 to 10,000 (Bivins *et al*. 2020). A recent preprint suggested that evaluating total RNA overestimated the number of intact viruses within wastewaters (Wurtzer *et al*. 2021). Enveloped viruses are considered less stable in the environment than non-enveloped viruses, such as human enteric viruses (e.g. norovirus), typically transmitted via the fecal-oral route and associated with waterborne transmission (Casanova and Weaver 2015). These observations align with recent opinions from water microbiologists and wastewater professionals (Maal-Bared *et al*. 2020) that wastewater does not appear to be a significant transmission route for SARS-CoV-2. Furthermore, presence of RNA in a sample is insufficient to infer the magnitude of the risk of waterborne transmission via wastewater or environmental waters.

Despite caveats associated with using RNA concentration for risk assessment, several studies have conducted quantitative microbial risk assessment (QMRA) for SARS-CoV-2 transmission from exposure to wastewater via oral or inhalation routes (Kumar *et al*. 2021b; Yang *et al*. 2020; Dada and Gyawali 2021; Gholipour *et al*. 2021; Shutler *et al*. 2021; Zaneti *et al*. 2021).

QMRA models are inherently limited by assumptions and uncertainties, including SARS-CoV-2 shedding rates and durations, the persistence of infectious SARS-CoV-2 in wastewater and wastewater aerosols, dilution rates in wastewater collection systems and ratios of RNA to infectious viruses (the models assumed ranges from 1000 to 1 and 29 to 1). Furthermore, each model uses a SARS-CoV-1 dose-response model (Watanabe *et al*. 2010) and some apply this inhalation dose-response model to other routes of exposure such as oral ingestion (Zaneti *et al*. 2021). There is also uncertainty surrounding morbidity ratios among those infected. While, multi-pathway risk assessments have not been conducted for wastewater exposures, QMRAs of multiple exposure routes among health care workers indicated the dominance of aerosol exposures as risk drivers (Mizukoshi *et al*. 2021).

Several risk analyses have also raised concerns regarding wastewater-impacted surface waters (Kumar *et al*. 2021b; Yang *et al*. 2020). Shutler *et al*. (2021), conducted a relative risk analysis of countrywide surface waters, calculating concentrations in receiving rivers after a sewage spill, highlighting situations where high infection rates among the wastewater-producing population and low dilution rates in the environment could result in ‘infectious doses’ of SARS-CoV-2 RNA >40 GC/100 mL in river water, which is reflective of sewage impacted waterways, but does not account for RNA to infectious virus ratios. Kumar *et al*. (2021b) estimated that per event infection risks from incidental ingestion of recreational water can range from 10^−5.84^ to 10^−2.61^ for swimming and fishing, respectively. Yang *et al*. (2020) combined these approaches and estimated per-exposure infection risks ranging from 10^−12^ to 10^−10^ across various inhalation scenarios. Across the three analyses, improved understanding of virus fate, transport and viability, and relevant exposure routes were highlighted as limitations.

Given the frequent detection of SARS-CoV-2 RNA in wastewater combined with the rare observations of infectious SARS-CoV-2 in feces/urine, the transmission of COVID-19 via wastewater is possible. However, the limited persistence of infectious SARS-CoV-2 in wastewater, and the well-established inhalation exposure route suggests the most probable wastewater-associated transmission scenario is fecal aerosols generated from newly-produced wastewater escaping to air via defective building plumbing as implicated previously for SARS-CoV-1 (McKinney, Gong and Lewis 2006). However, based on the current evidence, we assert that fecal-oral transmission of SARS-CoV-2 associated with wastewater is likely to be low compared to well-documented person-to-person transmission via respiratory droplets/aerosols. This assertion is largely premised on the failure to isolate infectious SARS-CoV-2 from wastewater or environmental waters in two peer-reviewed studies totalling 19 samples (Rimoldi *et al*. 2020; Westhaus *et al*. 2021). Furthermore, the low ratios of infectious SARS-CoV-2 to RNA in clinical samples, the limited persistence of infectious SARS-CoV-2 in environmental waters, as observed during three studies, and the efficacy of most WWTPs in virus reduction act as barriers to substantially reduce risks. SARS-CoV-2 infection risks from untreated and treated wastewaters and wastewater-impacted environmental waters are likely lower than other fecally-excreted pathogens such as norovirus and hepatitis A virus. Existing water quality regulations, biosafety protocols and procedures, which have been designed for waterborne pathogens, along with masks as recommended by the CDC (https://www.cdc.gov/coronavirus/2019-ncov/lab/lab-biosafety-guidelines.html), are sufficient to ensure the safety of the public and wastewater professionals (Brisolara *et al*. 2021).

Definitive conclusions about risk from wastewater exposures are constrained by the limited sample size of the research performed to date. Negative results, which are critical to establish upper bounds of risk (e.g. 0 in 20 is not equivalent to 0 in 1000), are less likely to be published due to bias against such results (Anonymous 2020). Additional empirical observations of infectious SARS-CoV-2 in wastewater or environmental waters, including negative results if available, are needed. Given the finite resources available for responding to the COVID-19 pandemic, possible transmission routes must be examined, considering their probability of contributing to disease. Researchers should continue to exercise caution and communicate the uncertainty and assumptions of their studies when leveraging models based on limited empirical evidence particularly during a global pandemic when these results can be misinterpreted.
